# Supplementary figures and images for: An efficient mixture of deep and machine learning models for COVID-19 diagnosis in chest X-ray images
Source: PLoS One. 2020 Nov 17;15(11):e0242535. doi: 10.1371/journal.pone.0242535 (PMC7671547; doi:10.1371/journal.pone.0242535)

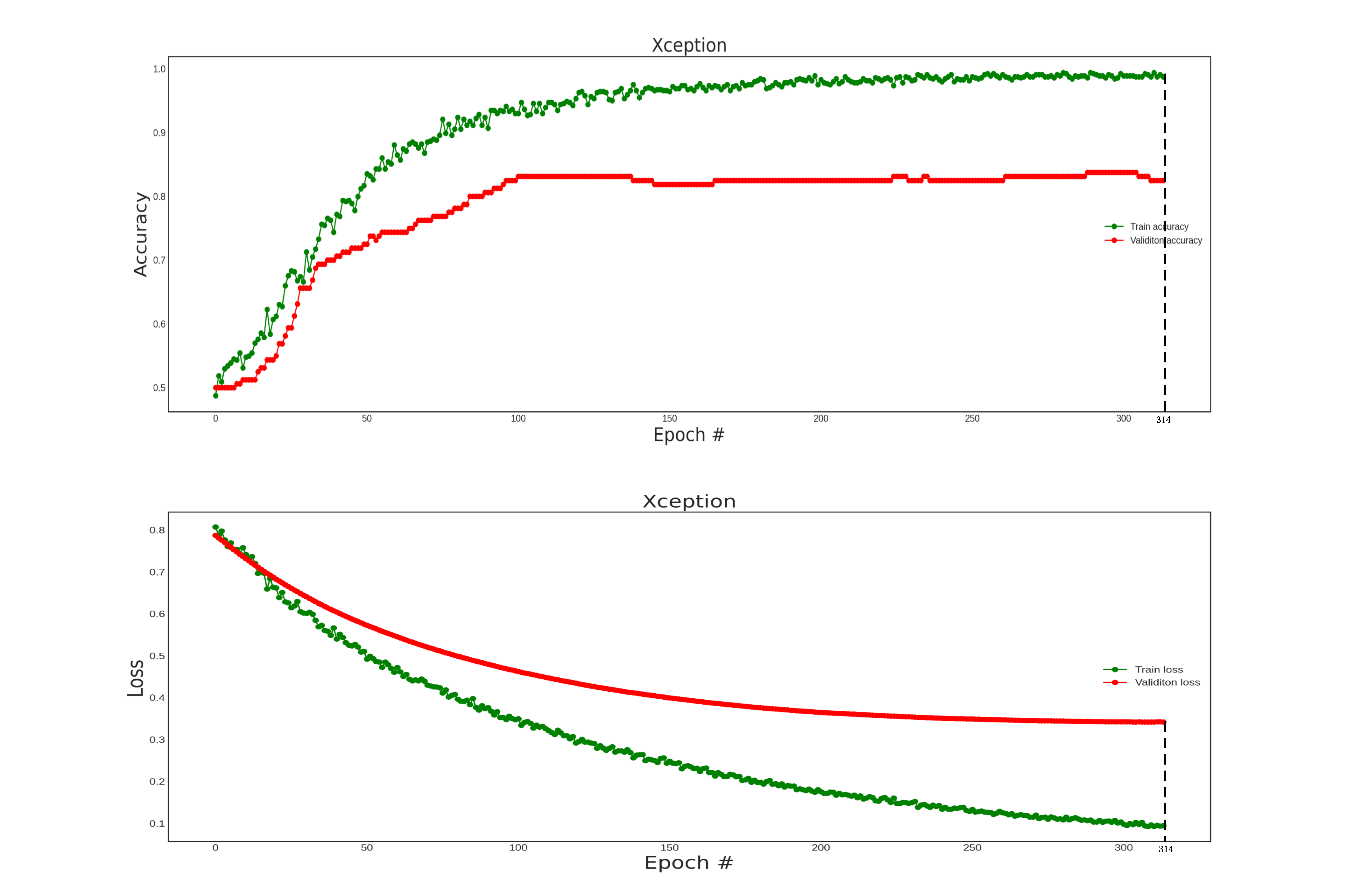


**S1 Fig.** **Train/Validition Accuracy and Train/Validition Loss curve of Xception model.**

Supplement: S1 Fig — (DOCX) [file pone.0242535.s001.docx]
