# Supplementary material for: An efficient mixture of deep and machine learning models for COVID-19 diagnosis in chest X-ray images
Source: PLoS One. 2020 Nov 17;15(11):e0242535. doi: 10.1371/journal.pone.0242535 (PMC7671547; doi:10.1371/journal.pone.0242535)
Supplement: S1 Table — (DOCX) [file pone.0242535.s002.docx]

**S1 Table. The average (AVR) and standard deviation (STD) of the five models run three times.**

| **Model** | **SEN%** | **SPE%** | **PRE%** | **ACC%** | **F1 %** | **AUC%** |
| --- | --- | --- | --- | --- | --- | --- |
| VGG16 run1 | 91.24 | 99.38 | 99.21 | 95.64 | 91.23 | 95.31 |
| VGG16 run2 | 91.97 | 98.76 | 98.44 | 95.64 | 95.10 | 95.36 |
| VGG16 run3 | 91.97 | 98.76 | 98.44 | 95.64 | 95.10 | 95.36 |
| **AVR of VGG16** | **97.73** | **98.97** | **98.70** | **95.64** | **93.81** | **95.34** |
| **STD of VGG16** | **0.34** | **0.29** | **0.36** | **0.00** | **1.82** | **0.02** |
| InceptionV3 run1 | 91.97 | 98.76 | 98.44 | 95.64 | 95.1 | 95.58 |
| InceptionV3 run2 | 92.7 | 98.76 | 98.45 | 95.57 | 95.49 | 95.83 |
| InceptionV3 run3 | 92.7 | 98.76 | 98.45 | 95.97 | 95.49 | 95.83 |
| **AVR of InceptionV3** | **92.46** | **98.76** | **98.45** | **95.72** | **95.36** | **95.75** |
| **STD of InceptionV3** | **0.34** | **0.00** | **0.00** | **0.17** | **0.18** | **0.12** |
| ResNet50 run1 | 86.13 | 95.65 | 94.4 | 91.28 | 90.08 | 90.89 |
| ResNet50 run2 | 89.78 | 96.27 | 95.35 | 93.29 | 92.48 | 93.03 |
| ResNet50 run3 | 91.97 | 95.03 | 94.03 | 93.62 | 92.99 | 93.5 |
| **AVR of ResNet50** | **89.29** | **95.65** | **94.59** | **92.73** | **91.85** | **92.47** |
| **STD of ResNet50** | **2.41** | **0.51** | **0.56** | **1.03** | **1.27** | **1.14** |
| Xception run1 | 97.81 | 99.38 | 99.26 | 98.66 | 98.53 | 98.59 |
| Xception run2 | 95.62 | 99.38 | 99.24 | 97.65 | 97.4 | 97.5 |
| Xception run3 | 95.62 | 99.38 | 99.24 | 97.65 | 97.4 | 97.5 |
| **AVR of Xception** | **96.35** | **99.38** | **99.25** | **97.99** | **97.78** | **97.86** |
| **STD of Xception** | **1.03** | **0.00** | **0.01** | **0.48** | **0.53** | **0.51** |
| DenseNet121 run1 | 91.97 | 98.76 | 98.44 | 95.64 | 95.1 | 95.36 |
| DenseNet121 run2 | 91.24 | 98.14 | 97.66 | 94.97 | 94.34 | 94.32 |
| DenseNet121 run3 | 90.51 | 98.14 | 97.64 | 94.63 | 93.94 | 94.58 |
| **AVR of DenseNet121** | **91.24** | **98.35** | **97.91** | **95.08** | **94.96** | **94.75** |
| **STD of DenseNet121** | **0.60** | **0.30** | **0.37** | **0.42** | **0.48** | **0.44** |
