# Supplementary material for: An efficient mixture of deep and machine learning models for COVID-19 diagnosis in chest X-ray images
Source: PLoS One. 2020 Nov 17;15(11):e0242535. doi: 10.1371/journal.pone.0242535 (PMC7671547; doi:10.1371/journal.pone.0242535)
Supplement: S2 Table — (DOCX) [file pone.0242535.s003.docx]

**S2 Table. The details of the five pre-trained convolutional neural network models.**

| Network Name | Input size | No. of layers | No. of parameters in Million |
| --- | --- | --- | --- |
| VGG16 | 224×224×3 | 41 | 138 |
| InceptionV3 | 299×299×3 | 824 | 23.2 |
| ResNet50 | 224×224×3 | 177 | 25.6 |
| Xception | 299×299×3 | 170 | 22.9 |
| DenseNet121 | 224×224×3 | 708 | 20 |
