# Supplementary material for: An efficient mixture of deep and machine learning models for COVID-19 diagnosis in chest X-ray images
Source: PLoS One. 2020 Nov 17;15(11):e0242535. doi: 10.1371/journal.pone.0242535 (PMC7671547; doi:10.1371/journal.pone.0242535)
Supplement: S3 Table — (DOCX) [file pone.0242535.s004.docx]

**S3 Table. Time comparison of the transfer learning and the proposed method (seconds)**

| Network Name | Time for transfer learning method  (Pre-trained model) | Time for proposed method  (Deep feature extraction) |
| --- | --- | --- |
| VGG16 | 571 | 39 |
| InceptionV3 | 151 | 44 |
| ResNet50 | 627 | 41 |
| Xception | 337 | 42 |
| DenseNet121 | 311 | 44 |
