# Supplementary material for: An efficient mixture of deep and machine learning models for COVID-19 diagnosis in chest X-ray images
Source: PLoS One. 2020 Nov 17;15(11):e0242535. doi: 10.1371/journal.pone.0242535 (PMC7671547; doi:10.1371/journal.pone.0242535)
Supplement: S4 Table — (DOCX) [file pone.0242535.s005.docx]

**S4 Table. Five different models combined with five different machine learning classifiers confusion matrix**

| Method | Confusion Matrix | | |  | | Method | Confusion Matrix | |  |  |
| --- | --- | --- | --- | --- | --- | --- | --- | --- | --- | --- |
|  | Predict Class | | |  |  |  | Predict Class | |  |  |
|  | C | | N |  |  |  | C | N |  |  |
| VGG16 + SVM | **129** | | **8** | C | True Class | InceptionV3 + SVM | **136** | **1** | C | True Class |
|  | **1** | | **160** | N |  |  | **2** | **159** | N |  |
| VGG16 + RF | **132** | | **5** | C |  | InceptionV3 + RF | **132** | **5** | C |  |
|  | **4** | | **157** | N |  |  | **3** | **158** | N |  |
| VGG16 + DT | **131** | | **6** | C |  | InceptionV3 + DT | **129** | **8** | C |  |
|  | **8** | | **153** | N |  |  | **3** | **158** | N |  |
| VGG16 + AdaBoost | **133** | | **4** | C |  | InceptionV3 + AdaBoost | **128** | **9** | C |  |
|  | **5** | | **156** | N |  |  | **5** | **156** | N |  |
| VGG16 + Bagging | **134** | | **3** | C |  | InceptionV3 + Bagging | **135** | **2** | C |  |
|  | **2** | | **159** | N |  |  | **2** | **159** | N |  |
| ResNet50 + SVM | **120** | | **17** | C | True Class | DenseNet121 + SVM | **132** | **5** | C | True Class |
|  | **16** | | **145** | N |  |  | **1** | **160** | N |  |
| ResNet50 + RF | **133** | | **4** | C |  | DenseNet121 + RF | **124** | **13** | C |  |
|  | **8** | | **153** | N |  |  | **1** | **160** | N |  |
| ResNet50 + DT | **124** | | **13** | C |  | DenseNet121 + DT | **131** | **6** | C |  |
|  | **10** | | **15** | N |  |  | **4** | **157** | N |  |
| ResNet50 +AdaBoost | **121** | | **16** | C |  | DenseNet121+ AdaBoost | **131** | **6** | C |  |
|  | **13** | | **148** | N |  |  | **4** | **157** | N |  |
| ResNet50 + Bagging | **132** | | **5** | C |  | DenseNet121 + Bagging | **130** | **7** | C |  |
|  | **6** | | **155** | N |  |  | **1** | **160** | N |  |
| Xception + SVM | **136** | **1** | | C | True Class |  |  |  |  |  |
|  | **1** | **160** | | N |  |  |  |  |  |  |
| Xception + RF | **134** | **3** | | C |  |  |  |  |  |  |
|  | **3** | **158** | | N |  |  |  |  |  |  |
| Xception + DT | **123** | **4** | | C |  |  |  |  |  |  |
|  | **7** | **154** | | N |  |  |  |  |  |  |
| Xception + AdaBoost | **122** | **15** | | C |  |  |  |  |  |  |
|  | **9** | **152** | | N |  |  |  |  |  |  |
| Xception+ Bagging | **135** | **2** | | C |  |  |  |  |  |  |
|  | **1** | **160** | | N |  |  |  |  |  |  |

C, COVID-19 cases; N, Normal cases.
